# Multicenter Validation of Natural Language Processing Algorithms for the Detection of Common Data Elements in Operative Notes for Total Hip Arthroplasty: Algorithm Development and Validation

**DOI:** 10.2196/38155

**Published:** 2022-08-31

**Authors:** Peijin Han, Sunyang Fu, Julie Kolis, Richard Hughes, Brian R Hallstrom, Martha Carvour, Hilal Maradit-Kremers, Sunghwan Sohn, VG Vinod Vydiswaran

**Affiliations:** 1 Department of Computational Medicine and Bioinformatics University of Michigan Ann Arbor, MI United States; 2 Department of Artificial Intelligence and Informatics Mayo Clinic Rochester, MN United States; 3 Department of Orthopedic Surgery University of Michigan Ann Arbor, MI United States; 4 Department of Internal Medicine and Epidemiology University of Iowa Iowa City, IA United States; 5 Departments of Orthopedic Surgery Mayo Clinic Rochester, MN United States; 6 Department of Learning Health Sciences Medical School University of Michigan Ann Arbor, MI United States; 7 School of Information University of Michigan Ann Arbor, MI United States

**Keywords:** total hip arthroplasty, natural language processing, information extraction, model transferability

## Abstract

**Background:**

Natural language processing (NLP) methods are powerful tools for extracting and analyzing critical information from free-text data. MedTaggerIE, an open-source NLP pipeline for information extraction based on text patterns, has been widely used in the annotation of clinical notes. A rule-based system, MedTagger-total hip arthroplasty (THA), developed based on MedTaggerIE, was previously shown to correctly identify the surgical *approach*, *fixation*, and *bearing surface* from the THA operative notes at Mayo Clinic.

**Objective:**

This study aimed to assess the implementability, usability, and portability of MedTagger-THA at two external institutions, Michigan Medicine and the University of Iowa, and provide lessons learned for best practices.

**Methods:**

We conducted iterative test-apply-refinement processes with three involved sites—the development site (Mayo Clinic) and two deployment sites (Michigan Medicine and the University of Iowa). Mayo Clinic was the primary NLP development site, with the THA registry as the gold standard. The activities at the two deployment sites included the extraction of the operative notes, gold standard development (Michigan: registry data; Iowa: manual chart review), the refinement of NLP algorithms on training data, and the evaluation of test data. Error analyses were conducted to understand language variations across sites. To further assess the model specificity for *approach* and *fixation*, we applied the refined MedTagger-THA to arthroscopic hip procedures and periacetabular osteotomy cases, as neither of these operative notes should contain any *approach* or *fixation* keywords.

**Results:**

MedTagger-THA algorithms were implemented and refined independently for both sites. At Michigan, the study comprised THA-related notes for 2569 patient-date pairs. Before model refinement, MedTagger-THA algorithms demonstrated excellent accuracy for *approach* (96.6%, 95% CI 94.6%-97.9%) and *fixation* (95.7%, 95% CI 92.4%-97.6%). These results were comparable with internal accuracy at the development site (99.2% for *approach* and 90.7% for *fixation*). Model refinement improved accuracies slightly for both *approach* (99%, 95% CI 97.6%-99.6%) and *fixation* (98%, 95% CI 95.3%-99.3%). The specificity of *approach* identification was 88.9% for arthroscopy cases, and the specificity of *fixation* identification was 100% for both periacetabular osteotomy and arthroscopy cases. At the Iowa site, the study comprised an overall data set of 100 operative notes (50 training notes and 50 test notes). MedTagger-THA algorithms achieved moderate-high performance on the training data. After model refinement, the model achieved high performance for *approach* (100%, 95% CI 91.3%-100%), *fixation* (98%, 95% CI 88.3%-100%), and *bearing surface* (92%, 95% CI 80.5%-97.3%).

**Conclusions:**

High performance across centers was achieved for the MedTagger-THA algorithms, demonstrating that they were sufficiently implementable, usable, and portable to different deployment sites. This study provided important lessons learned during the model deployment and validation processes, and it can serve as a reference for transferring rule-based electronic health record models.

## Introduction

### Background

Natural language processing (NLP) methods are powerful tools for extracting information from textual data and are widely applied in medical informatics research [1]. NLP approaches transform unstructured free-text clinical notes into a structured and codified format, thereby reducing human effort on chart reviews in large population-based studies [2-5]. Previous studies have demonstrated that NLP can be an alternative to manual abstraction in many applications, including deidentification, classification, and extraction of medical concepts (eg, clinical symptoms, diagnoses, and medications), semantic modifiers (eg, negation and severity), and temporality information (eg, present vs past; [6,7]). In addition, high-quality NLP approaches applied to real-world data can facilitate clinical registry participation and analysis [8] to further advance clinical research, policy, and surveillance efforts [6,9,10].

In prior research, Wyles et al [11] developed an NLP system to extract common data elements related to total hip arthroplasty (THA) from the operative notes in electronic health records (EHRs). This NLP system contains 3 separate algorithms aimed at capturing the operative *approach*, *fixation* method, and *bearing surface* categories [11,12]. The infrastructure of the NLP system was an open-source NLP pipeline, MedTaggerIE [13], which was developed using an open-source unstructured information management architecture–based information extraction framework [14]. MedTaggerIE contains the following three components: keyword lists (ie, domain-based keywords and short phrases, including wildcard regular expressions), classification rules (ie, regular expression-based patterns to derive the predicted label), and normalization (eg, a standardized form of any THA-related clinical concept). The classification rules take ≥1 regular expression as the input value to extract relevant information. The extracted concepts are normalized to the expected targets as output values. As keywords and phrases containing clinical information can be directly defined by subject matter experts (eg, orthopedic surgeons), the pipeline separates task-specific NLP knowledge engineering from the generic-domain NLP. The final system (referred to as *MedTagger-THA*) was evaluated on 250 THA procedures performed at the Mayo Clinic and demonstrated high accuracy in identifying the abovementioned 3 data elements [11]. The authors found MedTagger-THA to be a promising alternative to the current gold standard of manual chart review for identifying common data elements from orthopedic operative notes [11].

Although typically, the transferability of informatics tools across sites is poor [15] unless explicitly designed for, this data element extraction task is inherently portable across different sites. This is because the development site and the deployment sites (1) share common keywords for *approach* and *fixation* and (2) have common rules to classify *approach* and *fixation*. Some examples of such common rules include labeling “cement femur” and “uncemented shell” as “hybrid” and no “cement” mentions to indicate “uncemented.” However, prior studies have not broadly evaluated whether existing systems, when applied across multiple institutions with heterogeneous EHR systems, are sufficiently implementable (ie, whether the system can be deployed at a different site), usable (ie, whether the system can be easily modified and refined by local users), and portable (ie, whether the system can achieve sufficiently similar results after refinement). Prior studies have shown that significant effort is required for users to apply existing NLP systems [16]. In the context of multi-institutional collaboration, studies have indicated various administrative and implementation challenges such as data privacy; workforce expertise; and the maturity of location extract, transform, and load (ETL) processes [17]. For example, clinical NLP algorithms are often difficult to assess in different hospital settings because of patient confidentiality and difficulties in technology transfer [18]. In addition, the performances of clinical NLP systems, as well as clinical practice and workflows, often vary across institutions and source data [19,20], which results in differences in documentation styles in EHRs [21]. The clinical note structures and languages used within notes can be very different across institutions because of both syntactic variation and semantic variation in the text [21], highlighting the importance of correctly identifying sections [21,22] and semantic lexicon construction for extracting and encoding clinical information from EHRs to achieve semantic interoperability in developing NLP systems [23]. Therefore, to achieve better portability, all these factors must be considered when applying an NLP algorithm developed from one institution to another. In most cases, customization is necessary to achieve a desirable performance and further improve portability.

### Objectives

To assess and improve the implementability, portability, and usability of MedTagger-THA, we performed a pilot study to establish an efficient pipeline for transferring MedTagger-THA to 2 external institutions (Michigan Medicine and the University of Iowa) to provide lessons learned for best practices. This study included both common generic processes (eg, task definition, exchanging NLP resources, and training and evaluation) and site-specific processes. Specifically, we established the infrastructure to run MedTagger-THA, including accessing the electronic surgical notes, security clearance for implementation of the MedTagger software tool kit, and running and refining MedTagger-THA. MedTagger-THA algorithms were implemented and refined independently for both sites. At Michigan, we evaluated whether MedTagger-THA can accurately extract information on surgical *approach* and *fixation* from operative notes using the Michigan Arthroplasty Registry Collaborative Quality Initiative (MARCQI) registry as the gold standard. We assessed the out-of-box (prerefinement) validation performances and postrefinement performances on the extraction of *approach* and *fixation*. Finally, we assessed the specificity of these 2 data elements’ extraction using periacetabular osteotomy (PAO) and hip arthroscopy cases. As there was no existing arthroplasty registry at the Iowa site, manual chart review was used as the gold standard. We conducted a standardized gold standard development process, which included retrieving operative notes, developing annotation guidelines, and performing corpus annotation. We then used the gold standard to refine and evaluate the MedTagger-THA system for all three data elements—surgical *approach*, *fixation*, and *bearing surface*.

## Methods

### System Deployment of MedTagger

**Figure 1:**
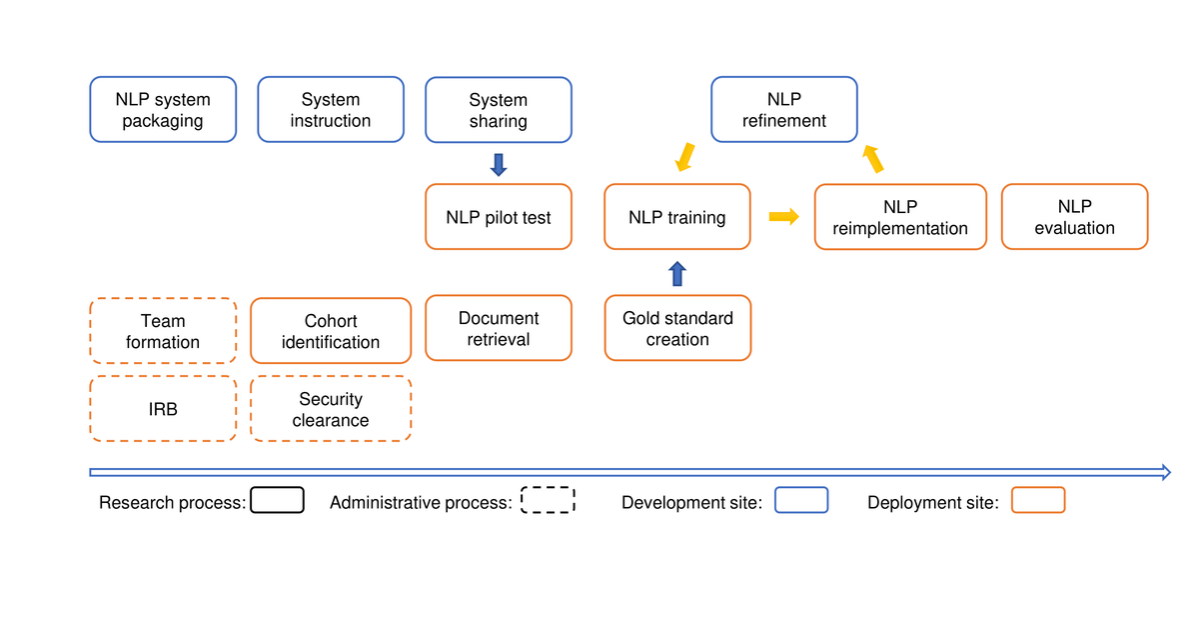
Overview of the NLP deployment and evaluation process. IRB: institutional review board; NLP: natural language processing.

MedTagger deployment was an iterative test-apply-refinement process involving close collaboration among sites (Figure 1). There were three involved sites: a development site (the site that developed the initial MedTagger-THA system, Mayo Clinic, shown in blue boxes) and 2 deployment sites (Michigan Medicine and the University of Iowa, shown in orange boxes). The initial step was to form an interdisciplinary study team with diverse backgrounds and expertise in orthopedics, information technology, informatics, and epidemiology. Once the team was established, the process was kicked off with several important administrative activities, including institutional review board (IRB) approval and system security clearance.

In addition to the administrative process, research activities were initiated simultaneously. System preparation and packaging were the initial steps at the development site. These steps focused on ascertaining whether the system was usable and interoperable at the deployment site. The NLP system contained two components: (1) a generic MedTagger framework (eg, sentence annotator, tokenizer, and part-of-speech tagger) and (2) MedTagger-THA algorithms (keyword lists and classification rules) that were developed and distributed separately from the main program. This architecture design allows THA algorithms to be easily plugged into the main program for better customizability. Therefore, the initial process was to separate the MedTagger-THA algorithms from the main program in MedTagger for distribution purposes. Following that, the next steps were to prepare the deployment site instructions, which included specifying the input text format (eg, rtf, xml, or plain text), preprocessing instructions, system directories, and system-level instructions and requirements: (1) operating system compatibility (PC, MAC, and Linux), (2) software and packages (Java 1.8), and (3) license (Apache version 2.0). Finally, for code exchange, we used the software development and version control platform Git.

### Michigan Site Process

#### Overview

The MARCQI is a group of orthopedic surgeons and medical professionals dedicated to improving the quality of care for patients undergoing hip and knee replacement procedures at Michigan Medicine. The consortium improves the quality of care by addressing variations in patient outcomes related to hip and knee joint replacement surgery [24]. THA cases were abstracted at Michigan Medicine and entered into the MARCQI data repository, including the date of surgery; laterality (left or right); and surgical *approach*, *fixation*, and *bearing surface*. In this study, the MARCQI registry was considered the gold standard to evaluate the automated algorithms. The surgical *approach* documented in the MARCQI included “Anterior,” “Anterolateral,” “Posterior,” and “Transtrochanteric.” The *fixation* methods included “Cemented,” “Uncemented,” “Hybrid,” and “Reverse Hybrid.” The *bearing surface* materials included “Ceramic-on-polyethylene,” “Metal-on-polyethylene,” and “Dual Mobility.”

We extracted the operative notes for elective and conversion primary THA performed between January 1, 2014, and April 30, 2019, from the Epic-based Michigan Medicine EHR system. As the *bearing surface* was captured by catalog numbers of implants used and not by notes abstraction, we only assessed the accuracy, precision, recall, and *F*_1_-score of the algorithms on *approach* and *fixation*. All 95% CIs were obtained using the procedure by Agresti and Coull [25].

In addition to THA, PAO and arthroscopy procedures are also conducted in Michigan Medicine and are sometimes applied to patients with THA. As these surgical procedures have some common features (such as *approach*), we believe it is necessary to assess the specificity of the algorithm to evaluate whether it is overly generalized. To assess the specificity of *fixation*, we applied the algorithms to PAO and hip arthroscopy cases as neither of these should have any kind of fixation that we were assessing. Hip arthroscopy cases were also used to assess the specificity of the algorithms for identifying the *approach* as arthroscopic hip procedures should not have an identified *approach*, as they were conducted through portals.

**Figure 2:**
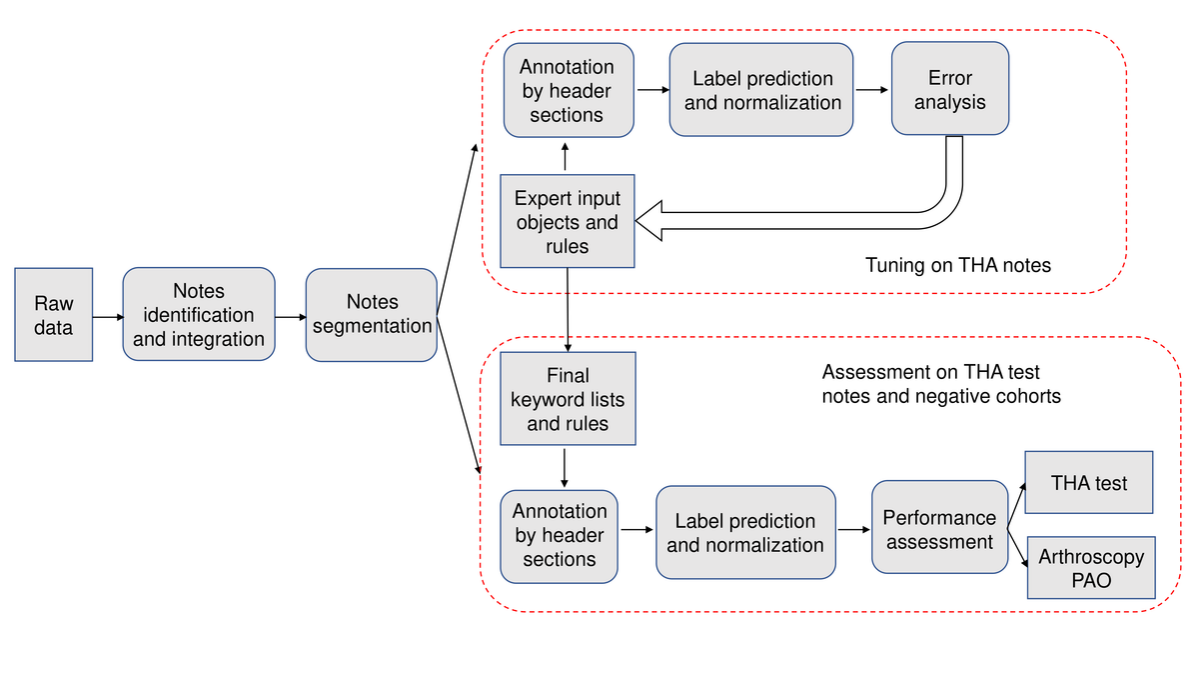
The workflow of the note-processing pipeline at the Michigan site. The rectangles represent the data and the rounded rectangles represent the process. PAO: periacetabular osteotomy; THA: total hip arthroplasty.

The *note-processing pipeline* that we established involved several steps (Figure 2).

#### Notes Identification and Integration

We first identified distinct patient-date pairs from THA notes, which represented procedures conducted on certain dates over specific individuals. For each patient, we ordered the notes by note documentation time and gathered all the notes that were within a 15-day interval as a note set for 1 operation. For 1 note set, we took the first documentation time to represent the patient’s procedure date. We then mapped patient-date pairs to the MARCQI data set. For patients with PAO and arthroscopy, we used the same 15-day window to integrate notes for unique patient-date pairs.

#### Notes Segmentation

For each unique patient-date pair, we first segmented the note sets by section headers. The section headers parsed from the THA notes are listed in Table S1 in Multimedia Appendix 1, which include concepts of preoperative diagnosis, procedure, findings, and implants. Among these headers, the section headers that were most likely to be semantically related to “procedures” (Table S2 in Multimedia Appendix 1) were predefined in the Michigan data. To refine the MedTagger-THA model using Michigan data, we first randomly split the data set into training (80%) and test (20%) sets based on unique patients. As the MARCQI only began to collect *fixation* data in 2017, THA notes before 2017 were excluded from these analyses.

#### Annotation by Header Sections

For each unique patient-date pair, the *approach* and *fixation* keywords were extracted from all relevant sections. The initial *approach* and *fixation* keywords were predefined using the keyword lists published previously [11]. As defined in the study by Wyles et al [11], “The assertion of each concept includes certainty (i.e., positive, negative, and possible) along with the person who experienced the event (i.e., the patient or someone else, such as husband, child, etc.), whereas temporality identifies the timing of an event (i.e., historical or present).” Concept with “positive” certainty, “present” temporality, and the “patient” who experienced the event is the concept of interest.

#### Label Prediction and Normalization

Classification rules comprising regular expressions were applied to derive prediction labels. The initial classification rules have been published previously [11]. For *approach*, the labels included “Anterior,” “Anterolateral,” “Posterior,” and “Transtrochanteric.” For *fixation*, the labels included “Cemented,” “Hybrid,” “Uncemented,” and “Reverse Hybrid.” The prediction labels also included two special conditions—if no annotation was given by any section, the final prediction would be “missing,” and if multiple annotations were given but were not the same, the final prediction would be “ambiguous.” For both the training and test sets, we applied MedTagger-THA [11] to extract the *approach* and *fixation* and evaluated their out-of-box performance.

#### Error Analysis

We then worked with the MARCQI abstraction professional to resolve the misclassifications, missing predictions, and ambiguous predictions in the training data set. We iteratively tuned the MedTagger-THA model [26] by adding keywords to the *approach* and *fixation* keyword lists and modifying the classification rules until the model performance could not be improved on the training data set. The test data set was not used during the refining process. After the refining process, we obtained the updated keyword lists and classification rules (Table S3 in Multimedia Appendix 1). Thus, in the following text, the refined MedTagger-THA obtained is referred to as MedTagger-THA-Michigan.

#### Assessment of THA Test Notes

We assessed the performance of MedTagger-THA-Michigan on the test data set. We further performed an error analysis on the test data set to analyze the limitations of the model. Finally, we evaluated the specificity of *approach* and *fixation* extraction from PAO and hip arthroscopy cases. Figure 2 shows the workflow of the Michigan identification pipeline.

### Iowa Site Process

We concurrently deployed the system at the University of Iowa. The gold standard corpus for the evaluation of the NLP system was established through a standard corpus annotation process [27]. A trained nurse abstractor manually reviewed 100 operative reports randomly sampled from known THA procedures between January 1, 2009, and December 31, 2016, from Iowa’s Epic-based EHRs. Questions regarding the abstracted data were resolved upon consultation with a physician with content expertise. Chart review was conducted using the same concept definition as that based on the total joint arthroplasty registry; in addition to *approach* and *fixation*, data collection included *bearing surface* classified into four categories: metal-on-polyethylene, ceramic-on-polyethylene, metal-on-metal, and ceramic-on-ceramic. The gold standard data set was equally split into 2 subsets of 50 training instances and 50 test instances. We followed an iterative training and refining process [26] to evaluate and refine the NLP algorithms. Briefly, the prototype system, MedTagger-THA, was applied to the training data. Error cases were manually reviewed by a team of researchers at Iowa with experience in informatics and clinical documentation to identify key errors or themes leading to missing or misclassified results. The keywords were manually curated through an iterative refining process until all major issues were resolved.

### Ethics Approval

The study was approved by the IRBs at both the University of Michigan (HUM00143841) and the University of Iowa (201903205).

## Results

### Michigan Site Results

For THA notes, 2304 unique patients with 2569 patient-date pairs were mapped to the MARCQI registry data set. From the PAO notes and arthroscopy notes, 398 and 523 patient-date pairs were extracted, respectively. For *approach* and *fixation*, the out-of-box external validation of the MedTagger-THA algorithms demonstrated excellent accuracy (surgical *approach*: 96.6%, 95% CI 94.6%-97.9%; *fixation*: 95.7%, 95% CI 92.4%-97.6%; Tables 1 and 2).

**Table 1:** Out-of-box performance of MedTagger-total hip arthroplasty (THA) for surgical approach: comparison of the gold standard (registry data) and notes classified by MedTagger-THA in the training and test data.^a^

Gold standard		MedTagger-THA, n (%)				
		Anterior	Anterolateral	Posterior	Ambiguous	Missing inference
**Training data (n=2062)**						
	Anterior	261 (12.7)	0 (0)	2 (0.1)	1 (0)	0 (0)
	Anterolateral	0 (0)	1 (0)	2 (0.1)	0 (0)	1 (0)
	Posterior	4 (0.2)	2 (0.1)	1737 (84.2)	1 (0)	50 (2.4)
**Test data (n=507)**						
	Anterior	68 (13.4)	0 (0)	0 (0)	0 (0)	0 (0)
	Anterolateral	0 (0)	1 (0.2)	0 (0)	0 (0)	0 (0)
	Posterior	0 (0)	1 (0.2)	421 (83)	0 (0)	15 (3)
	Transtrochanteric	0 (0)	0 (0)	0 (0)	0 (0)	1 (0.2)

^a^Accuracy: 96.6% (95% CI 94.6%-97.9%); precision: 99.8% (95% CI 98.7%-100%); recall: 96.6% (95% CI 94.6%-97.9%); *F*_1_-score: 98.2% (95% CI 96.5%-99.1%).

**Table 2:** Out-of-box performance of MedTagger-total hip arthroplasty (THA) for fixation: comparison of the gold standard (registry data) and notes classified by MedTagger-THA in the training and test data.^a^

Gold standard		MedTagger-THA, n (%)			
		Cemented	Hybrid	Uncemented	Ambiguous
**Training data (n=1053)**					
	Cemented	0 (0)	1 (0.1)	0 (0)	0 (0)
	Hybrid	1 (0.1)	76 (7.2)	3 (0.3)	17 (1.6)
	Uncemented	0 (0)	29 (2.8)	925 (87.8)	1 (0.1)
**Test data (n=256)**					
	Cemented	0 (0)	0 (0)	0 (0)	0 (0)
	Hybrid	0 (0)	23 (9)	2 (0.8)	5 (2)
	Uncemented	0 (0)	4 (1.6)	222 (86.7)	0 (0)

^a^Accuracy: 95.7% (95% CI 92.4%-97.6%); precision: 95.7% (95% CI 92.4%-97.6%); recall: 95.7% (95% CI 92.4%-97.6%); *F*_1_-score: 95.7% (95% CI 92.4%-97.6%).

The classification errors, ambiguous cases, and missing inferences are listed in Table 3. Classification errors for *approach* occurred when (1) the notes in one section contained mentions for a different *approach*, whereas the mentions for the correct *approach* were missing; (2) the mentions for a different *approach* were extracted from sections other than “procedure and findings”; and (3) the section of “procedure and findings” contained many different mentions for *approach*. Ambiguous cases occurred when mentions for the correct *approach* were extracted from notes related to “procedures and findings,” and different *approach* mentions were also extracted from other sections for a single surgery. Missing inferences occurred when the mentions for *approach* were missing in the notes or when the mentions were misspelled. Common classification errors for *fixation* occurred when the certainty of inference was incorrectly assessed. For example, for “non cemented stem,” the certainty was assessed as “positive” instead of “negative,” which resulted in an “Uncemented” *fixation* instance being misclassified as “Hybrid.” If the stem mentioned in the notes was not included in the predefined keyword list (eg, “femur”), a “Hybrid” instance was misclassified as “Uncemented,” or a “Cemented” instance was misclassified as “Hybrid.” “Hybrid” instances could also be misclassified as “Cemented” when “Cemented” was explicitly stated in the notes and a *Stem Concept* was noted, as the algorithm treated “Cemented” as a direct mention of *cemented fixation*. Similar situations were observed in ambiguous cases, where some sections misclassified “Hybrid” instances as “Cemented,” whereas others gave the correct classification. An “Uncemented” instance was inferred as a default *fixation* label when there was no mention of the “cement concept.” Therefore, if there was no mention of the “cement concept” explicitly, even if the surgery was “Cemented” or “Hybrid,” it was classified as “Uncemented.”

**Table 3:** Classification errors and ambiguous cases for approach and fixation in the Michigan data set.

Keyword	Classification error	Ambiguous cases	Missing
*Approach*	The mention of the correct *approach* was missing, although the mentions for other approaches existed. The notes in the “Complications” section contained mentions for a different *approach*, whereas the mentions for the correct *approach* were missing. Multiple different *approach* mentions were extracted from the same section, and the *approach* mentions that appeared more times were given priority.	Notes related to diagnosis sections but not the procedures contained different mentions of *approach*; for example, “Left hip osteoarthritis, abductor deficiency (sclerosed greater trochanter with chronic avulsion of gluteus medius)” was annotated as “anterolateral,” but the gold standard label was “posterior.” Notes related to “indications” contained hypothetical conditions; for example, “We offered her the option of anterior or posterior *approach* and she decided that an anterior *approach* was preferable.” was annotated as “posterior” instead of “anterior.”	Direct mentions of *approach* were not included in the keyword list; for example, “posterior THA^a^ precautions,” “APPROACH: Posterior,” and “posterolateral.” No mentions indicating the *approach* as the notes referred to previous incisions. Misspelling of the mentions led to unrecognition (eg, “shortrotators”).
*Fixation*	“Uncemented” was misclassified as “Hybrid” The note mentioned “non cement stem” but the certainty of the inference was positive for the *Cement Concept*.^b^ “Hybrid” was misclassified as “Uncemented”; for example, “femur” was not included in the stem keyword list, and no *Cement Concept* was mentioned in the notes. The surgeries were “Total Hip Replacement with Computer Navigation.” “Cemented” was misclassified as “Hybrid” as “femur” was not included in the stem keyword list, *Shell Concept*^b^ was also excluded. Only *Cement Concept* led to “Hybrid”; for example, “A polyethylene acetabular liner was cemented in using the trabecular metal acetabular revision system longevity, 0-degree face angle, 36-millimeter inner diameter VerSys Hip prosthesis standard neck offset size 11 was cemented into the femur.” “Hybrid” was misclassified as “Cemented” as “Cemented” was a direct mention and had priority over others; for example: “Total Hip Arthroplasty, cemented, Right Hip” was misclassified as “Cemented” In the notes, only the femoral canal is cemented.	For a single surgery note, some sections misclassified “Hybrid” as “Cemented” as “Cemented” was a direct mention of *Cement Concept* and had the highest priority over others; for example, “Total Hip Arthroplasty, cemented femoral stem” was misclassified as “cemented” instead of “Hybrid.”	Missingness in *fixation* was set to “Uncemented.”

^a^THA: total hip arthroplasty.

^b^Concept name.

After model refinement (Tables 4 and 5), the validation accuracies improved for both surgical *approach* and *fixation* (*approach*: 99%, 95% CI 97.6%-99.6% vs 96.6%; *fixation*: 98%, 95% CI 95.3%-99.3% vs 95.7%). Giving priorities to sections related to “procedures” reduced the ambiguous cases for *fixation* (from 5 to 2). For specificity assessment, we identified the *approach* mentioned in 11.1% (58/523) of patient-date pairs for the arthroscopy data set (specificity: 465/523, 88.9%). These false positives were mainly because of the keywords for the approach mentioned in the notes, such as “Hana table,” “anterior superior iliac spine,” or “tensor fascia lata,” although these mentions described positioning and portal placement. At times, arthroscopy was combined with PAO in a procedure, and the mentions for *approach* could be related to PAO. We did not identify any *fixation* mentioned in the PAO cohort or in the arthroscopy cohort (specificity 100%).

**Table 4:** *Approach* after refinement: comparison of the gold standard and notes classified by refined MedTagger-total hip arthroplasty (THA) in the Michigan test data set (N=507).^a^

Gold standard	MedTagger-THA-Michigan, n (%)				
	Anterior	Anterolateral	Posterior	Ambiguous	Missing inference
Anterior	68 (13.4)	0 (0)	0 (0)	0 (0)	0 (0)
Anterolateral	0 (0)	1 (0.2)	0 (0)	0 (0)	0 (0)
Posterior	0 (0)	0 (0)	434 (85.6)	0 (0)	3 (0.6)
Transtrochanteric	0 (0)	0 (0)	1 (0.2)	0 (0)	0 (0)

^a^Accuracy: 99% (95% CI 97.6%-99.6%); precision: 99.6% (95% CI 98.4%-100%); recall: 99% (95% CI 97.6%-99.6%); *F*_1_-score: 99.3% (95% CI 98%-99.8%).

**Table 5:** *Fixation* after refinement: comparison of the gold standard and notes classified by refined MedTagger-total hip arthroplasty (THA) in the Michigan test data set (N=256).^a^

Gold standard	MedTagger-THA-Michigan, n (%)			
	Cemented	Hybrid	Uncemented	Ambiguous
Cemented	0 (0)	0 (0)	0 (0)	0 (0)
Hybrid	1 (0.4)	26 (10.2)	1 (0.4)	2 (0.8)
Uncemented	0 (0)	1 (0.4)	225 (87.9)	0 (0)

^a^Accuracy: 98% (95% CI 95.3%-99.3%); precision: 98% (95% CI 95.3%-99.3%); recall: 98% (95% CI 95.3%-99.3%); *F*_1_-score: 98% (95% CI 95.3%-99.3%).

### Iowa Site Results

No registry data were available at the University of Iowa. Therefore, we performed a manual chart review of a total of 100 operative reports (50 training reports and 50 test reports) and tested the performance of MedTagger-THA on this data set for *approach* (Table 6), *fixation* (Table 7), and *bearing surface* (Table 8). Overall, the model achieved moderate-high performance on the training data, with the lowest performance observed for the *bearing surface* concept. Model refinement included modifying the default output for the *bearing surface* to match the case distribution of Iowa’s data and adding additional *liner*-related concepts (eg, A-class liner) to improve the sensitivity of the *fixation* category. After model refinement, the model achieved high performance for all three data elements: *approach* (100%, 95% CI 91.3%-100%), *fixation* (98%, 95% CI 88.3%-100%), and *bearing surface* (92%, 95% CI 80.5%-97.3%).

**Table 6:** *Approach*: comparison of the gold standard and notes classified by MedTagger-total hip arthroplasty (THA) in the University of Iowa data set (N=100).^a^

Gold standard			MedTagger-THA-Iowa, n (%)						Total, n (%)
			Anterior		Anterolateral		Posterior		
**Training data (n=50)**									
	Anterior	12 (24)		1 (2)		0 (0)		13 (26)	
	Anterolateral	0 (0)		0 (0)		0 (0)		0 (0)	
	Posterior	0 (0)		0 (0)		37 (74)		37 (74)	
**Test data (n=50)**									
	Anterior	14 (28)		0 (0)		0 (0)		14 (28)	
	Anterolateral	0 (0)		0 (0)		0 (0)		0 (0)	
	Posterior	0 (0)		0 (0)		36 (72)		36 (72)	

^a^Accuracy: 100% (95% CI 91.3%-100%); precision 100% (95% CI 91.3%-100%); recall: 100% (95% CI 91.3%-100%); *F*_1_-score: 100% (95% CI 91.3%-100%).

**Table 7:** *Fixation*: comparison of the gold standard and notes classified by MedTagger-total hip arthroplasty (THA) in the University of Iowa data set (N=100).^a^

Gold standard		MedTagger-THA-Iowa, n (%)				Total, n (%)
		Cemented	Hybrid	Uncemented		
**Training data (n=50)**						
	Cemented	0 (0)	0 (0)	0 (0)	0 (0)	
	Hybrid	0 (0)	1 (2)	0 (0)	1 (2)	
	Uncemented	0 (0)	0 (0)	49 (98)	49 (98)	
**Test data (n=50)**						
	Cemented	0 (0)	0 (0)	0 (0)	0 (0)	
	Hybrid	1 (2)	0 (0)	0 (0)	1 (2)	
	Uncemented	0 (0)	0 (0)	49 (98)	49 (98)	

^a^Accuracy: 98% (95% CI 88.3%-100%); precision: 98% (95% CI 88.3%-100%); recall: 98% (95% CI 88.3%-100%); *F*_1_-score: 98% (95% CI 88.3%-100%).

**Table 8:** *Bearing surface*: comparison of the gold standard and notes classified by MedTagger-total hip arthroplasty (THA) in the University of Iowa data set (N=100).^a^

Gold standard			MedTagger-THA-Iowa, n (%)									Total, n (%)
			MoP^b^		CoP^c^		MoM^d^		CoC^e^			
**Training data (n=50)**												
	MoP	25 (50)		1 (2)		1 (2)		0 (0)		27 (54)		
	CoP	0 (0)		17 (34)		0 (0)		0 (0)		17 (34)		
	MoM	0 (0)		0 (0)		0 (0)		0 (0)		0 (0)		
	CoC	0 (0)		6 (12)		0 (0)		0 (0)		6 (12)		
**Test data (n=50)**												
	MoP	20 (40)		2 (4)		0 (0)		0 (0)		22 (44)		
	CoP	0 (0)		26 (52)		0 (0)		0 (0)		26 (52)		
	MoM	0 (0)		0 (0)		0 (0)		1 (2)		1 (2)		
	CoC	0 (0)		1 (2)		0 (0)		0 (0)		1 (2)		

^a^Accuracy: 92% (95% CI: 80.5%-97.3%); precision: 92% (95% CI 80.5%-97.3%); recall: 92% (95% CI 80.5%-97.3%); *F*_1_-score: 92% (95% CI 80.5%-97.3%).

^b^MoP: metal-on-polyethylene

^c^CoP: ceramic-on-polyethylene.

^d^MoM: metal-on-metal.

^e^CoC: ceramic-on-ceramic.

## Discussion

### Principal Findings

In this study, we applied the MedTagger-THA algorithms developed at Mayo Clinic to the THA operative notes at Michigan Medicine and the University of Iowa. The algorithms were implementable, usable, and portable, with high performances at both deployment sites. Model refinements for major or recurring errors further improved the accuracy. In NLP reimplementation studies, refinement of the original model to “adapt” to the local health care system is important for the portability of the EHR models. We plan to validate MedTagger-THA in different hospital settings and EHRs and integrate these adapted models back into the original model. We expect that the continuous model refinement will further enhance portability.

We learned many important lessons from the NLP deployment and evaluation across different institutions. When assessing implementability, we encountered several workforce-related, institutional policy–related, and data infrastructure–related challenges and gaps. First, successful deployment and evaluation require at least three types of expertise: orthopedic domain knowledge of total joint arthroplasty, ETL skills, and expertise in NLP and model evaluation. We observed variable expertise at different sites and a strong need for multidisciplinary team science collaboration. Second, institutional policies have a significant impact on the time and effort related to the exchange of informatics resources. For example, the process of obtaining security clearances for sharing NLP systems to a locally secured environment could range from days to months depending on institutional policies. We also discovered a variation of strictness among institutions for sharing the NLP results for error analysis and refinement, suggesting the need for early planning and communication for multisite NLP research beyond just a multi-institutional IRB. The third aspect is the maturity of ETL and data infrastructure. There is substantial variation in institutional ETL processes and personnel training because of different data infrastructures. An institution with lower data infrastructure maturity would involve a manual abstraction process as an alternative, which can be a huge barrier for high-throughput NLP solutions. Specifically, the data infrastructure at Mayo Clinic is a centralized unified data platform, a duplication of the Epic Clarity table for handling various data retrieval requests in a central location. In contrast, Iowa has several decentralized enterprise data warehouses that require multiple ETL processes for data retrieval. Michigan maintains a separate research data warehouse for clinical and translational research, with a separate ETL pipeline to populate the warehouse with structured and free-text data. The aforementioned findings indicate the high complexity and dynamics of the multi-institutional EHR environment and suggest the need for a situated contextual understanding of multisite clinical NLP research.

When assessing usability and portability, there are some caveats in the process of NLP model refinement. We noticed that giving priorities to sections that related to “procedures” reduced the ambiguous cases. The headers of these sections may vary from site to site and require curation by medical experts to guarantee semantic interoperability. It is always possible to add curated keywords to the keyword list; however, these keywords may not be compatible with the original settings. For example, the negation algorithm was adopted from *ConText* [28]. “Posterior THA precautions” and “posterior THA” were considered “negated” in the original MedTagger-THA algorithms, as “precautions” is an indicator of “possible” instead of “positive” certainty according to *ConText* [28]. However, these mentions were indications of the posterior *approach* in Michigan’s data. We also changed the rules for identifying *fixation* better in Michigan’s data; however, we were not sure whether these changes would compromise the model performance at Mayo Clinic. These observations indicate the need to differentiate portable components of the model from institution-specific components that do not generalize well across institutions. Therefore, in the future refinement of MedTagger-THA, we suggest that a panel of medical experts and abstraction specialists from both the development site and validation and deployment sites should determine which changes can be incorporated into the original model for further distribution and better portability and which changes should be retained at the local validation site for institution-specific performance improvements.

We also noticed that *approach* and *fixation* were not unique mentions in THA notes. Keywords for the THA approach can be mentioned in other procedures, such as total knee arthroplasty, PAO, and arthroscopy, although those descriptions were not related to THA. As MedTagger-THA extracted information based on keyword mentions and rules defined by a series of regular expressions, we should acknowledge that the model should only be applied to THA notes. Therefore, before applying the MedTagger-THA model, it is necessary to filter out the non-THA operative notes. This process is relatively straightforward using text-based search and filtering, as the procedure names are usually explicitly mentioned in the “procedure” section.

MedTagger-THA algorithms are very useful for identifying THA-related data elements; however, they have several important limitations. MedTagger-THA was developed based on keywords and classification rules. Although we were able to extract keywords mentioned if the misspelled keywords were found during curation and training, future versions of MedTagger-THA should incorporate a validated spell check and correction model. In addition, MedTagger-THA cannot recognize hypothetical alternate treatment plans, such as whether the procedure was actually performed or merely documented as differentially discussed. MedTagger-THA links concepts by their locations in the texts (eg, *Cement Concept* close to *Stem Concept* means the stem is cemented) but cannot process the contextualized information (eg, 2 concepts were not related to each other). To solve these problems, we plan to conduct future research focusing on understanding the contextualized information when performing named entity recognition tasks using more advanced NLP techniques, such as methods based on machine learning, including deep learning models. Finally, for the Iowa site, the data for algorithm validation and refinement may be biased from the Iowa population of patients with THA because of the small sample size (n=100) and only one annotator being involved. Validation and refinement using small sample sizes may be valid in centers where clinical practice variability is low and thus, might increase accessibility to NLP-based tools where data infrastructural resources are limited or in development.

### Conclusions

In conclusion, MedTagger-THA algorithms were sufficiently implementable, usable, and portable to different deployment sites for *approach* and *fixation* identification from THA notes. *Bearing surface* identification may be subject to greater variability in clinical practice patterns and surgical devices. As expected, model refinement within unique institutional EHRs is useful for improving accuracy. This study underscores the importance of undertaking such model refinements in institutional settings and informs future implementation efforts to enhance transferability across institutions.

## Appendix

Multimedia Appendix 1 Section headers in operative notes, headers related to “procedures” and updated keyword lists, and classification rules for the total hip arthroplasty approach and fixation classification (Michigan).

## Abbreviations

EHR: electronic health record
ETL: extract, transform, and load
IRB: institutional review board
MARCQI: Michigan Arthroplasty Registry Collaborative Quality Initiative
NLP: natural language processing
PAO: periacetabular osteotomy
THA: total hip arthroplasty
