# Supplementary material for: Multicenter Validation of Natural Language Processing Algorithms for the Detection of Common Data Elements in Operative Notes for Total Hip Arthroplasty: Algorithm Development and Validation
Source: JMIR Med Inform. 2022 Aug 31;10(8):e38155. doi: 10.2196/38155 (PMC9475406; doi:10.2196/38155)
Supplement: Multimedia Appendix 1 [file medinform_v10i8e38155_app1.docx]

**Supplement Table 1.** Section headers in operative notes

| 001 Acetabular Shell:  002 Anticoagulation plan:  003 Atrial Lead:  004 Device:  005 Femoral Head:  006 Femoral Stem:  007 Incision care:  008 LV Lead:  009 Pathway plus:  010 Patient instructions:  011 Poly:  012 Post op pain control:  013 Pulmonary vein conduction assessment:  014 RV Lead:  015 poly:  016 dvt ppx:  017 2nd Assist:  018 3D-RENDERING WITH TRUS IMAGE PROCESSING AND FUSION:  019 4. Post implant instructions:  020 > Post Op Plan:  021 ACCESS:  022 ADDITIONAL ASSISTANTS:  023 ANESTHESIA:  024 ANORECTAL EXAM:  025 ASSESSMENT:  026 ASSISSTANT:  027 ASSIST:  028 ASSISTANT SURGEON:  029 ASSISTANT SURGEONS:  030 ASSISTANT(S):  031 ASSISTANT(s): Surgeon(s) and Role:  032 ASSISTANT:  033 ASSISTANTS:  034 ATTENDING ATTESTATION:  035 ATTENDING PHYSICIAN:  036 ATTENDING PHYSICIANS:  037 ATTENDING SURGEON:  038 ATTESTATION :  039 ATTESTATION:  040 Abandoned Hardware:  041 Access:  042 Active Hardware:  043 Addendum:  044 Anesthesia:  045 Antibiotics:  046 Anticoagulation:  047 Assistant Attestation:  048 Assistant SURGEON:  049 Assistant Surgeon:  050 Assistant:  051 Assisting surgeon:  052 Atrial Lead:  053 Attending Physician:  054 Attending surgeon:  055 Attestation:  056 BRADY PARAMETERS:  057 BRADYCARDIA SETTINGS:  058 BRIEF HISTORY AND INDICATIONS FOR PROCEDURE:  059 BRIEF HISTORY:  060 Baseline measurements:  061 Baseline pulmonary vein conduction study:  062 Biopsy locations were as follows:  063 Brady Parameters:  064 Brief Clinical History:  065 Brief Neurological Exam:  066 Brief Summary of Procedure Performed:  067 CCEP FELLOW/ASSISTANT:  068 CCEP FELLOW:  069 CCEP Fellow:  070 COMMENTS:  071 COMPLICATIONS:  072 COMPONENTS:  073 CONCLUSION:  074 CONCLUSIONS:  075 CONSENT AND TIME OUT:  076 CS Lead:  077 CYSTOSCOPY:  078 Cardiologists:  079 Cemented liner:  080 Cemented stem:  081 Cerebral angiography with left common carotid artery injection:  082 Cerebral angiography with left internal carotid artery injection:  083 Cerebral angiography with left subclavian artery injection:  084 Cerebral angiography with left vertebral artery injection:  085 Cerebral angiography with right common carotid artery injection:  086 Cerebral angiography with right internal carotid artery injection:  087 Cerebral angiography with right vertebral artery injection:  088 Comment:  089 Comments:  090 Complications:  091 Components:  092 Conclusion:  093 Conduit for Coronary Artery Bypass Grafting:  094 Contact:  095 DATE OF SURGERY:  096 DEFIBRILLATION THRESHOLD TEST:  097 DESCRIPTION OF OPERATIVE PROCEDURE:  098 DESCRIPTION OF PROCEDURE:  099 DESCRIPTION OF THE PROCEDURE:  100 DETAILED PROCEDURE AND FINDINGS:  101 DETAILS OF PROCEDURE:  102 DETAILS OF THE OPERATION:  103 DEVICES:  104 DIAGNOSES:  105 DISPOSITION:  106 DRAINS:  107 Date of Procedure:  108 Description of Procedure:  109 Description of procedure in detail:  110 Description of procedure:  111 Device Information:  112 Device Lead Data:  113 Dispo:  114 Disposition: Stable to recovery Procedure:  115 Drains:  116 EBL:  117 EQUIPMENT USED:  118 ESTIMATED BLOOD LOSS:  119 EXAM:  120 EXPLANTS:  121 Equipment use:  122 Estimated Blood Loss:  123 Estimated Blood loss:  124 Estimated blood loss:  125 Explantations of hardware:  126 Explanted Device:  127 Explanted Hardware:  128 FELLOW SURGEON:  129 FINDINGS AND DETAILS:  130 FINDINGS:  131 FIXATION:  132 FLUIDS:  133 FLUOROSCOPY TIME:  134 FOLLOW UP AND PLAN:  135 Fellow:  136 Findings and Operative Course:  137 Findings:  138 First Assist:  139 Fixation:  140 Fluids:  141 Follow-Up Plan:  142 Follow-up/Recommendations:  143 Following Procedures were Performed:  144 Freeze 1:  145 Freeze 2:  146 HARDWARE INFORMATION:  147 HISTORY:  148 Hardware removal:  149 Hardware:  150 Heart Transplant:  151 Heme:  152 IMPLANT FIXATION:  153 IMPLANTS:  154 IMPRESSION:  155 IMplants:  156 INDICATION FOR PROCEDURE:  157 INDICATION:  158 INDICATIONS FOR OPERATION:  159 INDICATIONS FOR PROCEDURE:  160 INDICATIONS FOR SURGERY:  161 INDICATIONS:  162 INSTRUMENTATION USED:  163 INSTRUMENTATION:  164 INTRAOPERATIVE FINDINGS:  165 IOPTH findings:  166 ISpecimens removed:  167 IV MEDICATIONS GIVEN BY DEPARTMENTAL PROTOCOL:  168 Implant:  169 Implants removed:  170 Implants:  171 In summary, the following was performed:  172 Indication for Procedure:  173 Indication:  174 Indications for Surgery:  175 Indications for procedure:  176 Indications:  177 Informed Consent Discussion:  178 Interventional Cardiologist:  179 LEAD PARAMETERS:  180 LEFT VENTRICULAR LEAD:  181 LUTS:  182 LV LEAD:  183 LV lead details:  184 LV lead:  185 LVAD Removal and ICD lead removal:  186 Left Ventricular Lead:  187 Left pulmonary vein potentials:  188 Left pulmonary veins:  189 Legend for prostate lesion location:  190 MEASURED DEVICE DATA:  191 MICROSURGICAL DETAILS:  192 MRI Findings:  193 Measured Device Data:  194 Measurements:  195 Medications Administered:  196 NEW MEDICATION POST PROCEDURE:  197 NEW MEDICATIONS POST-PROCEDURE:  198 New Medications:  199 OPERATION PERFORMED:  200 OPERATION(S) PERFORMED:  201 OPERATION/PROCEDURE:  202 OPERATION:  203 OPERATIONS PERFORMED:  204 OPERATIONS:  205 OPERATIVE FINDINGS:  206 OPERATIVE INDICATIONS:  207 OPERATIVE PROCEDURE:  208 OPERATIVE PROCEDURES AND FINDINGS:  209 OPERATIVE PROCEDURES:  210 OPERATIVE REPORT:  211 OPERATIVE TECHNIQUE:  212 OPERATORS:  213 Operation Performed:  214 Operation Performed: Procedure(s):  215 Operation performed:  216 Operative Procedure:  217 Operative procedure:  218 Our post-operative plan includes:  219 PLAN:  220 POST OP DIAGNOSIS:  221 POST-OPERATIVE DIAGNOSES:  222 POST-OPERATIVE:  223 POSTOP DIAGNOSES:  224 POSTOPERATIVE DIAGNOSES:  225 POSTOPERATIVE DIAGNOSIS(ES):  226 POSTOPERATIVE DIAGNOSIS:  227 POSTOPERATIVE PLAN:  228 POSTPROCEDURE DIAGNOSES:  229 PRE-OPERATIVE DIAGNOSES:  230 PRE-OPERATIVE DIAGNOSIS:  231 PREOP DIAGNOSES:  232 PREOP DIAGNOSIS:  233 PREOP LABS:  234 PREOP LUTS:  235 PREOPERATIVE DIAGNOSES:  236 PREOPERATIVE DIAGNOSIS (ES):  237 PREOPERATIVE DIAGNOSIS(ES):  238 PREOPERATIVE DIAGNOSIS:  239 PREOPERATIVE INDICATIONS:  240 PROCEDURAL SUMMARY:  241 PROCEDURE AND DETAILS:  242 PROCEDURE AND FINDINGS:  243 PROCEDURE DETAIL:  244 PROCEDURE DETAILS:  245 PROCEDURE NOTE:  246 PROCEDURE PERFORMED:  247 PROCEDURE SUMMARY:  248 PROCEDURE(S) PERFORMED:  249 PROCEDURE(S):  250 PROCEDURE:  251 PROCEDURE: Procedure(s):  252 PROCEDURES AND FINDINGS:  253 PROCEDURES PERFORMED:  254 PROCEDURES PERFORMED: Procedure(s):  255 PROCEDURES:  256 PROCEDURES: Left shoulder arthroscopy with:  257 PROGRAMMED PARAMETERS:  258 PROSTATE BIOPSY:  259 PROSTATE CRYOABLATION:  260 PT/OT:  261 Panel 1 (General Surgery):  262 Panel 1:  263 Panel 2 (Urology):  264 Panel 2:  265 Panel 3:  266 Parameters:  267 Participating surgeon(s): Surgeon(s):  268 Past Medical History:  269 Patient referred by:  270 Plan:  271 Planned Stent removal date:  272 Post Operative Diagnosis:  273 Post op plan:  274 Post procedure:  275 Post-Op Diagnosis Codes:  276 Post-Op Diagnosis/Findings:  277 Post-Op Diagnosis:  278 Post-Procedure Preliminary findings:  279 Post-ablation Electrophysiologic Study:  280 Post-operative Plan:  281 Postop Diagnosis:  282 Postop instructions:  283 Postoperative Care:  284 Postoperative Diagnosis:  285 Postoperative Plan:  286 Postoperative diagnosis:  287 Postoperative plan:  288 Pre-Op Diagnosis:  289 Pre-operative Diagnosis:  290 Pre-operative diagnosis:  291 Preop Diagnosis:  292 Preop Exam Neurological Exam:  293 Preoperative Diagnosis:  294 Preoperative diagnosis:  295 Preparation for Initiation of Cardiopulmonary Bypass:  296 Prior biopsy pathology:  297 Prior biopsy:  298 Procedure Details:  299 Procedure Performed:  300 Procedure and Findings:  301 Procedure and findings:  302 Procedure in Detail:  303 Procedure performed:  304 Procedure(s) (LRB):  305 Procedure(s) Performed:  306 Procedure(s):  307 Procedure:  308 Procedures performed:  309 Procedures:  310 Prosthetic Device/Implant:  311 Pulmonary Vein Ablation:  312 Pulmonary vein assessment after PV ablation:  313 Pulses: Pre op: Post Op:  314 RA lead:  315 RECOMMENDATION:  316 RECOMMENDATIONS FOR FOLLOW UP AND PLAN:  317 RECOMMENDATIONS FOR FOLLOW-UP AND PLAN:  318 RECOMMENDATIONS FOR FOLLOWUP AND PLAN:  319 RECOMMENDATIONS FOR POSTOP CARE:  320 RECOMMENDATIONS/PLAN:  321 RECOMMENDATIONS:  322 RECTAL SWAB CULTURE:  323 RESIDENT SURGEON(S):  324 RESIDENT SURGEON:  325 RESIDENT SURGEONS:  326 RESIDENT/FELLOW SURGEON(S):  327 RESIDENT:  328 RESIDENTS:  329 RIGHT ATRIAL LEAD:  330 RIGHT VENTRICULAR LEAD:  331 ROCK Summary:  332 ROCKS Summary:  333 RV lead:  334 Recommendations/Plan:  335 Recommendations:  336 Referring physician:  337 Right Ventricular Lead:  338 Right pulmonary vein potentials:  339 Right pulmonary veins:  340 SPECIMEN:  341 SPECIMENS OBTAINED:  342 SPECIMENS REMOVED:  343 SPECIMENS:  344 STAFF:  345 SUMMARY OF FINDINGS:  346 SUMMARY OF PROCEDURE AND FINDINGS:  347 SUMMARY:  348 SURGEON(S) AND ROLE(S):  349 SURGEON, ASSISTANT AND SUPERVISOR:  350 SURGEON:  351 SURGEONS:  352 SURGEONS: :  353 SURGERY:  354 SURGICAL FINDINGS:  355 SURGICAL PROCEDURE:  356 Second Assistant:  357 Sedation:  358 Separation from Cardiopulmonary Bypass:  359 Sheaths:  360 Specimen (Bacteriological, Pathological or other):  361 Specimen:  362 Specimens Removed:  363 Specimens removed:  364 Specimens removed: Implant:  365 Specimens removed: Specimens removed:  366 Specimens removed: pecimens removed:  367 Specimens removed:Specimens removed:  368 Specimens:  369 Surgeon(s) and Role:  370 Surgeon(s):  371 Surgeon(s): Surgeon(s):  372 Surgeon:  373 Surgeon: Surgeon(s):  374 Surgeons Narrative:  375 Surgeons and Role:  376 Surgical Attending Addendum:  377 TACHY PARAMETERS:  378 TACHY-THERAPY SETTINGS:  379 TIME OUT:  380 TOTAL IV FLUIDS:  381 TRANSRECTAL ULTRASOUND:  382 TREATMENT PLANNING:  383 TRUS:  384 TUBES AND DRAINS:  385 Tachyparameters:  386 The target lesion location was:  387 Title of Operation:  388 Tourniquet Time:  389 Urine Output:  390 anesthesia:  391 antibiotics:  392 assist:  393 beginning  394 complications:  395 components:  396 contact:  397 diagnoses:  398 dispo:  399 drains:  400 exam:  401 findings:  402 fluids:  403 hardware:  404 implants:  405 indications:  406 operative indications:  407 plan:  408 procedure:  409 procedures:  410 sedation:  411 surgeon(s):  412 surgeon:  413 surgery: |
| --- |

**Supplement Table 2.** Section headers related to “procedures”

| 199 OPERATION PERFORMED:  200 OPERATION(S) PERFORMED:  201 OPERATION/PROCEDURE:  202 OPERATION:  203 OPERATIONS PERFORMED:  204 OPERATIONS:  207 OPERATIVE PROCEDURE:  209 OPERATIVE PROCEDURES:  213 Operation Performed:  214 Operation Performed: Procedure(s):  215 Operation performed:  216 Operative Procedure:  217 Operative procedure:  240 PROCEDURAL SUMMARY:  241 PROCEDURE AND DETAILS:  242 PROCEDURE AND FINDINGS:  243 PROCEDURE DETAIL:  244 PROCEDURE DETAILS:  245 PROCEDURE NOTE:  246 PROCEDURE PERFORMED:  247 PROCEDURE SUMMARY:  248 PROCEDURE(S) PERFORMED:  249 PROCEDURE(S):  250 PROCEDURE:  251 PROCEDURE: Procedure(s):  252 PROCEDURES AND FINDINGS:  253 PROCEDURES PERFORMED:  254 PROCEDURES PERFORMED: Procedure(s):  255 PROCEDURES:  298 Procedure Details:  299 Procedure Performed:  300 Procedure and Findings:  301 Procedure and findings:  302 Procedure in Detail:  303 Procedure performed:  304 Procedure(s) (LRB):  305 Procedure(s) Performed:  306 Procedure(s):  307 Procedure:  308 Procedures performed:  309 Procedures: |
| --- |

**Supplement Table 3.** Updated keyword lists and classification rules for THA approach and fixation classification (Michigan)

|  | Approach | Fixation |
| --- | --- | --- |
| Keyword lists | Add “approach: anterior”, “approach: anterolateral”, “approach: posterior”, “approach: posterolateral”, “posterolateral”, “posterior hip arthroplasty precautions”, and “posterior tha” | Add “femur” to the keyword list for [Stem Concept] |
| Classification rules | Gave notes in sections related to “procedures” the higher priority to determine the final prediction. | - Remove “cement” from direct mention for cemented. - Modify rule for “cemented”: ([Stem Concept] AND [Cement Concept]) AND ([Liner/Shell Concept] AND [Cement Concept]). - Modify rule for “Hybrid”: if the direct mention of “Hybrid” existed, or ([No Shell Concept] AND [Cement Concept]), or ([Stem Cement Concept] AND [No Liner Cement Concept]), or ([Stem Cement Concept] AND [No Shell Cement Concept]). |
| Misspelled vocabulary | Add “shortrotators” |  |
| Context rules | Remove negation conditions for “precautious” | Add negation for “non cemented stem” |
